# Differential Transcript Expression and Alternative RNA Splicing Patterns to Differentiate Focal vs. Generalized-Onset Seizures

**DOI:** 10.1007/s12035-025-05110-1

**Published:** 2025-06-11

**Authors:** Rashi Verma, Katie L. Bullinger, Andrea Pearson, Monica Dhakar, Emine Guven, Elham Amini, Roger P. Simon, Robert Meller

**Affiliations:** 1 Neuroscience Institute, Morehouse School of Medicine, 720 Westview Drive SW, Atlanta, GA USA; 2 Department of Neurology, Emory University, Atlanta, GA USA; 3 Institute of Translational Genomic Medicine, Morehouse School of Medicine, Atlanta, GA USA

**Keywords:** RNA-sequencing, Transcriptional usage, Epilepsy, Focal, Generalized, Seizure, Diagnosis

## Abstract

Retrospective diagnosis of a seizure type is pivotal for effective management and treatment of epilepsy. Previously, we demonstrated that RNA signatures could discriminate between non-epileptic spells and epileptic seizures. Here, we investigate the utility of alternative RNA splicing to distinguish generalized versus focal epileptic seizures. Blood samples were collected at baseline, 4–6 h post-seizure, and at discharge from 27 patients undergoing video-electroencephalogram (vEEG) monitoring at the Emory University Hospital. Epileptologists determined seizure classification through vEEG data review. RNA was extracted, sequenced, and analyzed for RNA expression and transcript usage. Classification models were generated to distinguish between patients who had a focal or generalized seizure. The study shows transcriptomic profile changes following EEG-verified focal and generalized seizures. Compared to baseline, focal seizure exhibits limited changes in transcriptomic expression 4–6 h post-seizure and discharge samples. In contrast, generalized seizures demonstrated a broader transcript response, with 74 differentially expressed transcripts at 4–6 h and 70 at discharge. The changes were also evident across different time points between focal and generalized seizure. The study for the first time described the landscape of isoform switching in seizure type. Notably, significant isoform switching without differences in gene expression was observed. We identified 2689 isoform switches linked to 1249 genes among which 742 genes were sensitive to nonsense-mediated mRNA decay (NMD). Significant switches were observed in genes such as CORO1C, ZBTB44, SNHG1, and RPS17. Notably, we also observed novel isoforms, including CD300 (MSTRG.26116.1), RNF216 (MSTRG.52862.7), and RN7SL1 (MSTRG.17010.3) which exhibited significant switching, revealing potential new regulators of gene expression. Differentially expressed transcripts were utilized as classifiers for machine learning (ML) modeling using random forest (rf) and radial support vector machine (rSVM) algorithms, achieving ~ 83% accuracy in classifying generalized seizures, and multivariate adaptive regression splines (mars) algorithm achieving 100% accuracy in identifying focal seizure events. Our findings of blood transcript expression changes, including isoform switch analysis, underscore the potential of blood-based transcriptome analysis for retrospectively distinguishing seizure types and identifying biomarkers for epilepsy management.

**Supplementary Information:**

The online version contains supplementary material available at 10.1007/s12035-025-05110-1.

## Introduction

Epileptic seizures are episodic events of abnormal brain activities ranging from brief lapses in awareness to full temporary loss of consciousness, and full-body convulsions which are characterized by a sudden, and unpredictable onset [1]. A differential diagnosis of a retrospective seizure is based on patient history and clinical manifestation, that makes diagnosis of seizure type quite challenging [2]. While video-electroencephalography (vEEG) is the gold standard that provides comprehensive monitoring of brain activity, it is costly, time-intensive, and limited to specialized epilepsy centers for seizure identification [3]. These limitations restrict its use in retrospective seizure type classification, particularly in emergency settings where rapid treatment decisions are crucial.

Several putative biomarkers (prolactin, neuron specific enolase, βS-100) have been identified to distinguish psychogenic non-epileptic seizures (PNES) from epileptic seizures, thereby improving initial diagnosis [4–6]. However, once an epileptic seizure is confirmed, no reliable biomarkers exist to further classify it as focal or generalized seizure—an essential distinction for guiding treatment. Although automated seizure detection methods are useful for real-time monitoring, they are of limited utility when patients present after a seizure, often leading to restrictive or suboptimal treatment choices. Furthermore, access to specialized expertise, variability in symptom description among patients, and the ongoing need for refining diagnostic criteria further compound these challenges.

The misdiagnosis of seizure type at onset is a concern for two reasons. First, some therapies may be more effective for specific seizure types, as such delayed diagnosis leads to delayed therapy initiation and subsequently reduced responsiveness to treatment [7]. Second, inappropriate anti-seizure medication prescriptions given to a misdiagnosed patient not only impact the individual’s quality of life but also incur significant costs for both the patient and the healthcare system. Thus, accurate determination of seizure type is pivotal for effective management and treatment of epileptic seizures.

To this end, we explored the differential splicing and transcript-level expression signatures of RNA extracted from whole blood samples obtained from patients undergoing vEEG monitoring for epilepsy. We further investigated the functional impact of transcript isoform switching and discovered several novel transcripts unique to specific seizure types. Our approach offers a comprehensive understanding of seizure types, aiding in real-time monitoring, retrospective diagnosis, and the development of biomarkers for personalized anti-epileptic treatment strategies.

## Methods

### Ethics Approval, Study Setting, and Patient Consent

The study (Protocol #1536255; dated-01/14/2020) was reviewed and approved by the Institutional Review Board (IRB) of Morehouse School of Medicine (MSM) and Emory University, Atlanta. The study adhered to the ethical guidelines detailed in our previous publication [8]. Patients were recruited from the adult Epilepsy Monitoring Unit (EMU) at Emory University Hospital’s epilepsy center between July 2020 and July 2023. Patients who were seizure free for 5 days prior to admission and who provided informed consent were included in this study. Individuals were excluded if they had taken an investigational drug treatment during the preceding 3 months or had undergone gene or cellular therapy (Suppl. Figure 1) [9–11]. Written informed consent was obtained from all participants.

### Sample Collection, RNA Isolation, and Processing

Whole blood samples (3 mL) were collected and maintained based on guideline outline in our previous publication [8]. Demographic and clinical information pertaining to the participants, such as age, sex, racial background, medication regimens, drug screening results, usage of anti-seizure medications, history of epilepsy, and details of prior treatments, were extracted from their respective medical records for comprehensive evaluation. RNA was extracted from blood samples using the pre-AnalytiX RNA extraction kit (Qiagen). Subsequent RNA library construction, quantification, and sequencing were performed following the established protocol described in our previous publication [8].

### Seizure Classification

Patients were monitored on a 24 h continuous vEEG for the duration of their hospital stay. Video and electroencephalography data were analyzed by the study epileptologist (KLB) for seizure semiology and seizure localization (focal vs. generalized). Seizures were classified as focal or generalized as per the International League Against Epilepsy (ILAE) seizure classification guidelines [12]. Samples were grouped according to classification of the individual event captured as focal seizure without impaired awareness (FA), focal seizure with impaired awareness (FIA), focal to bilateral tonic clinic (FTC), generalized seizure with impaired awareness (GIA), and generalized tonic clinic (GTC). Seizures were classified according to initial onset localization. For example, FIA and FTC seizures were classified as “focal” whereas GTC or GIA seizures were classified as “generalized”.

### Sequencing Data, Mapping, and Annotation

Data were converted from bam files to fastq format, trimmed with TrimGalore (v0.6.4) (https://github.com/FelixKrueger/TrimGalore) and aligned to the human reference genome (GRCh38) using STAR (v2.7.3a) and Bowtie2 (v2.3.5.1) [13, 14]. The resulting bam files were used for assembly and annotation of transcripts, followed by normalization to transcript per million (TPM). Transcript quantification, encompassing both known and novel transcripts, was obtained from the output GTF file generated through StringTie2 (v2.2.1) [15]. Subsequently, a count matrix was compiled utilizing a custom python script (PrepDE.py) (full scripts available at https://github.com/rob-meller/ and https://github.com/Vermarashi/).

### Transcript Expression Analysis

The transcript expression was calculated using the transcript count matrix coupled with phenotypic information. Statistical analysis was performed using the edgeR-limma framework in R (v4.3) [16, 17]. Transcripts were filtered and TMM normalized. Incorporating sample ID into the model controlled for random effects. The two-fold change (2-FC) values were computed for each comparison, with expression data subjected to Bayesian smoothing of standard errors. Differential isoform expression was determined based on 2-FC and a false discovery rate (FDR)-corrected* p* value < 0.05. R packages such as VennDiagram, ggplot, and EnhancedVolcano were used to construct Venn diagrams, PCA, and volcano plots, respectively (full scripts available at https://github.com/Vermarashi/).

### Isoform Switching and Its Functional Consequence

Differential isoform switching or transcript usage analysis was conducted using IsoformSwitchAnalyzeR package in R [18]. A cutoff value of 0.1 for the differential isoform fraction (dIF) was applied to identify significant changes, excluding genes with only one isoform [18]. The “extractSplicingSummary” and “extractConsequenceSummary” functions were then used to evaluate the impact of these changes on splicing patterns and their functional consequences, respectively (full scripts available at https://github.com/Vermarashi/).

### RNA Interactome and Gene Ontology Enrichment Analysis

Differentially expressed transcript usage genes were identified and used for the construction of a network using Cytoscape (v3.10.1) [19]. This network gathered data from the STRING database, selecting interactions with a confidence score of ≥ 0.4 to construct a protein–protein interaction (PPI) network. Gene ontology analysis was then conducted to identify the functional role of genes identified between focal vs. generalized seizure; (i) paired with baseline, and (ii) across time points. For this, cytoscape plug-in “ClueGO” (v2.5.10), was employed, with enrichment significance set at *p* < 0.05 [20].

### Machine Learning (ML)-Driven Seizure Classification

The CPM count matrix was loaded into R as a csv file, and expression values filtered using lists of expressed transcripts between baseline and either 4–6 h focal seizure samples, or 4–6 h generalized seizure samples. We trained machine learning models [GLMnet (glm), random forest (rf), Naive Bayes (NB), radial support vector machine (rSVM), multivariate adaptive regression splines (mars), and decision tree (DT)] using data from focal seizure patients (*N* = 21) and tested the models on data from generalized seizure patients (*N* = 6). The tuning length was set to 10, with tenfold cross-validation applied to the models. A classifier model was used to classify the seizure type (no = baseline/yes = seizure), with AUC-ROC chosen as the evaluation metric. Likewise, we trained a second model on generalized seizure data and tested on focal seizure data using same parameters. The R package Caret and CaretEnsemble were used to run modeling [21] (full scripts available at https://github.com/Vermarashi/).

## Results

### Participant Enrollment

Out of 260 admissions, 258 patients were initially considered for the study. However, 136 were excluded based on inclusion criteria. Nineteen patients opted out. In all, 77 patients consented to participate, among which 36 patients did not experience seizures and three withdrew. Four samples were removed from analysis as events were determined to be non-epileptic, and one additional sample was removed as the seizure type remained unclear after vEEG analysis. Six samples failed for quality check (Suppl. Figure 1). A total of 27 sets of patient samples with either focal or generalized seizure as determined by EEG and chart review by a board-certified epileptologist were included in this study (Table 1).

**Table 1:** Patient data included in the study

*Type*	Baseline	4–6 h	Discharge
*Seizure*	27	26	24
*Generalized*	6 [5GTC + (1GIA*)]	6 [5GTC + (1GIA*)]	6 [5GTC + (1GIA*)]
*Focal*	21 [13 FIA + 7 FTC + (1 Subclinical*^#^)]	20 [12 FIA + 7 FTC + (1 Subclinical*^#^)]	18 [12 FIA + 5 FTC + (1 Subclinical*^#^)]

*GTC*, generalized tonic-clonic seizures; *GIA*, generalized absence seizures; *FTC*, focal tonic-clonic seizures; F*IA*, focal impaired awareness seizure. *Samples removed from subtype analysis. ^**#**^Focal subclinical electrographic seizure which is characterized by EEG-confirmed focal onset without associated clinical signs or symptoms, as defined by the American Clinical Neurophysiology Society (ACNS)

### Differential Expression Profiles Following Seizure Type Paired with Baseline

**Fig. 1:**
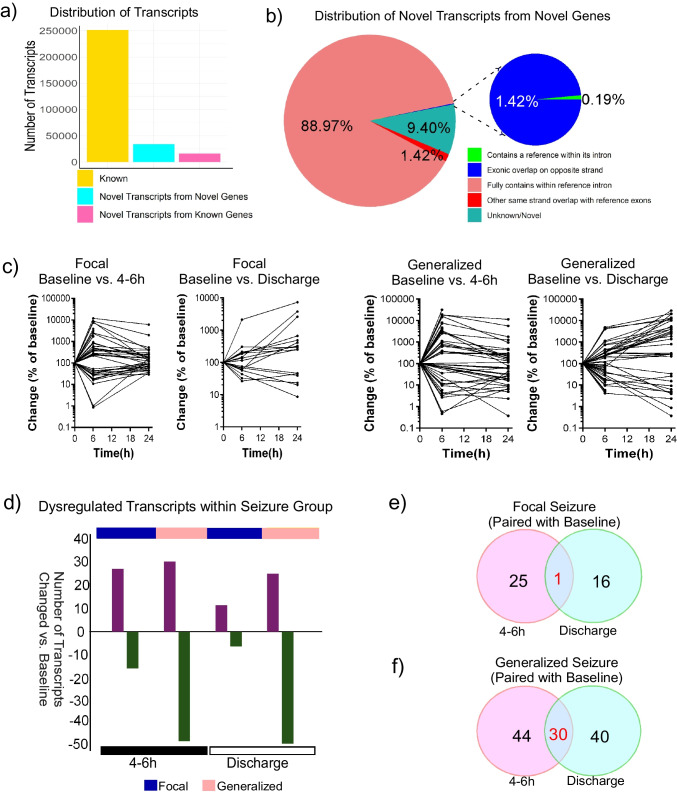
Temporal dynamics of differential transcript expression in focal and generalized seizures paired with baseline. **a** Bar graph of 50,081 novel transcripts within the GRCh38 genome. A total of 67.7% were associated with novel genes, and 32.3% with known genes. **b** Pie chart illustrates the categorization of novel transcripts among which 3191 novel isoforms from novel genes with no overlap with the reference genome. **c** Line graph of the mean transcript expression of differential expressed transcripts, normalized to the baseline value (100%). Data are separated based on the transcript lists of differentially expressed transcripts for each condition relative to the baseline samples. **d** Bar plots displayed the dysregulated transcripts (up and down) within the seizure groups with respect to baseline. The number of transcripts increase are represented in purple and decrease in dark green in generalized seizure. Interestingly, an increased transcriptional response was evident shortly after the focal seizure, while in generalized seizures, a more profound transcriptional response occurred at 4–6 h post-seizure but persisted till discharge. **e** Pie chart depicts consistent dysregulation of one transcript in focal seizure and **f** 30 transcripts in generalized seizure, indicating distinct temporal transcriptional responses and underlying molecular mechanisms between seizure types

We identified 50,081 novel transcripts in our dataset that aligned with the GRCh38 human genome, with 67.7% mapping to novel genes and 32.3% associated with known genes (Fig. 1a). Among these, 3191 novel isoforms derived from novel genes showed no overlap with the GRCh38 reference genome (Fig. 1b). We then analyzed differential transcript expression following generalized and focal seizures, comparing post-seizure samples to their respective baseline samples (Table 1). In patients with focal seizures, we identified 41 differentially expressed transcripts between baseline and 4–6 h post-seizure samples (26 upregulated, 15 downregulated) and 17 transcripts between baseline and discharge samples (11 upregulated, 6 downregulated). In contrast, patients with generalized seizures exhibited a more extensive transcriptomic response, with 74 differentially expressed transcripts between baseline and 4–6 h post-seizure samples (29 upregulated, 45 downregulated) and 70 transcripts between baseline and discharge samples (24 upregulated, 46 downregulated) (FDR-corrected *p* < 0.05, two-fold change) (Fig. 1c and d). When evaluating persistent transcriptomic changes, we identified one common dysregulated transcript in both the 4–6 h and discharge phases of focal seizures (Fig. 1e). In contrast, a more sustained transcript response was observed following generalized seizures, with 30 transcripts consistently dysregulated at both the 4–6 h and discharge time points compared to baseline (Fig. 1f).

### Differential Temporal Expression of Transcripts Following Focal or Generalized Seizures

**Fig. 2:**
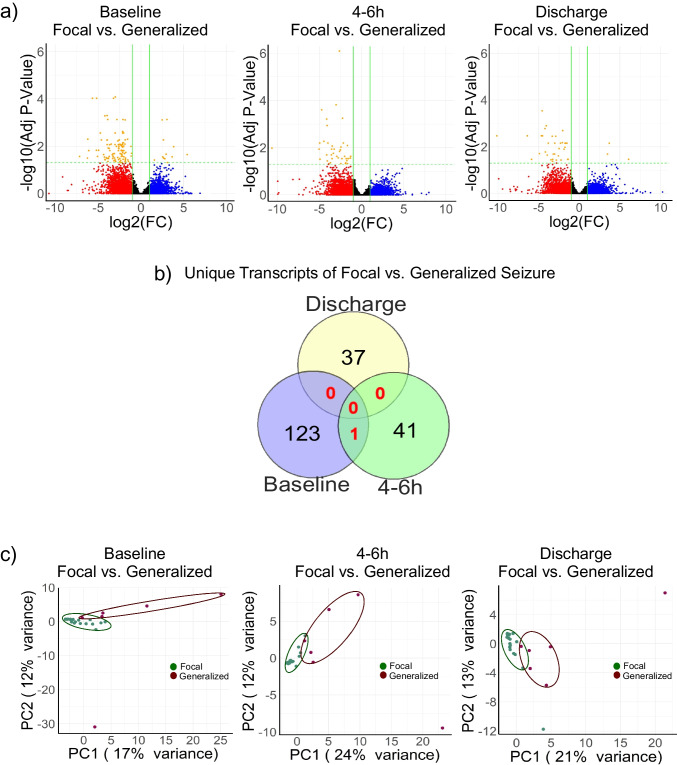
Characterization of novel transcripts and differential transcript expression in seizure patients. **a** Volcano plot showing differentially expressed transcript between focal and generalized seizure at baseline, 4–6 h post-seizure and discharge [black dots—not significant; blue dots—upregulated; red dots—downregulated; orange dots—significant for *p* value < 0.05 as well as twofold change]. **b** Venn diagram showing the unique transcripts in following seizure across time points. Note the lack of overlap between these clinical conditions suggesting different sets of transcripts show differential expression over time. **c** Principal component analysis (PCA) showing a distinct separation between focal and generalized seizures

We further analyzed transcript expression in patient samples of generalized and focal seizures at baseline, 4–6 h post-seizure, and at discharge (Table 1). The analysis showed 124 transcripts are significantly different between generalized and focal seizure patients at baseline, 42 at 4–6 h post-seizure, and 37 at discharge (FDR-corrected *p* < 0.05, 2-FC) (Fig. 2c). Interestingly only one transcript overlaps across the distinct time points emphasizing the dynamic nature of seizure-associated transcriptomic profiles (Fig. 2d). The distinct separation between conditions was discernible between focal and generalized seizures in PCA plots of the 4–6 h and discharge samples (24% and 21% variance, respectively) (Fig. 2e).

### Differential Transcript Usage Identified Across Seizure Types

**Fig. 3:**
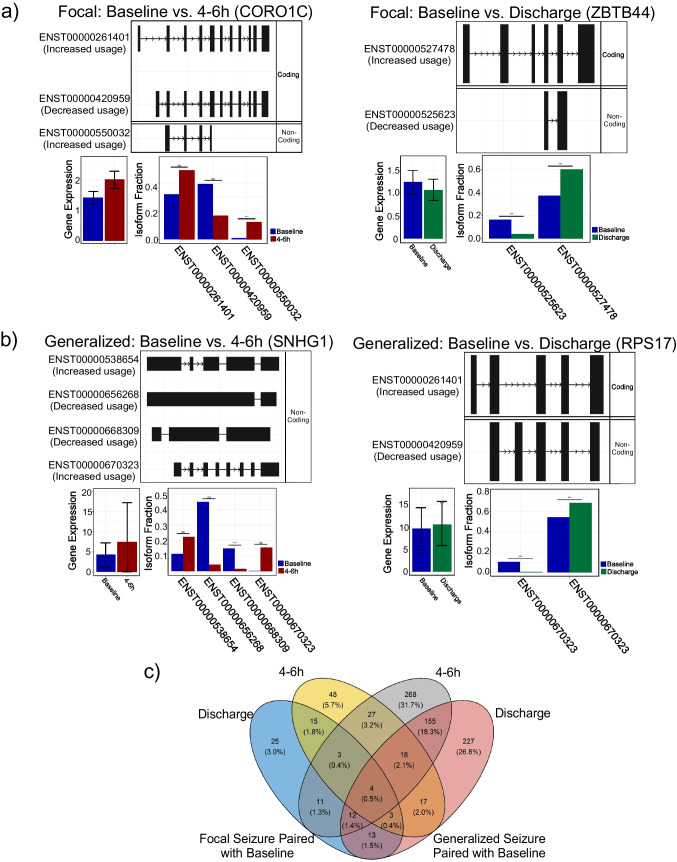
Top alternatively spliced genes following seizure type compared to baseline, based on q-values. **a** Focal seizures: CORO1 C (baseline vs. 4–6 h) and ZBTB44 (baseline vs. discharge) were identified as the top-ranked isoforms. **b** Generalized seizures: SNHG1 (baseline vs. 4–6 h) and RPS17 (baseline vs. discharge) emerged as the top-ranked isoforms. **c** Unique isoform switching events relative to baseline revealed that focal seizures exhibited 48 distinct switches at 4–6 h post-seizure and 25 at discharge, whereas generalized seizures showed 268 unique switches at 4–6 h and 227 at discharge, highlighting distinct molecular responses between the two seizure types

We assessed the differential use of transcript isoforms (isoform switching) using the R package IsoformSwitchAnalyzeR (|dIF|> 0.1) [18]. Isoform switching between focal and generalized seizure revealed 2748 isoforms linked to 2689 switching events in 1249 unique genes. Comparing post-seizure time points to baseline, CORO1C (*q* = 4.18 × 10^⁻^^32^) and ZBTB44 (*q* = 1.47 × 10^⁻^^20^) emerged as top switches in focal seizures (Fig. 3a), while SNHG1 (*q* = 3.26 × 10^⁻^^54^) and RPS17 (*q* = 2.14 × 10^⁻^^32^) were most significant in generalized seizures (Fig. 3b). The highest number of unique switching events was observed in generalized seizure (4–6 h post-seizure, 31.7%; discharge, 26.8%) as compared to focal seizure (4–6 h post-seizure, 5.7%; discharge, 3.0%). However, we also observed small but significant overlaps of common isoform switches following both seizure types (Fig. 3c).

We then focused on genes associated with significant differential isoform fractions across time points. We show examples of FECH, PPP1R18, and S100A9 as the topmost alternatively spliced genes between focal and generalized seizures in baseline, 4–6 h post-seizure, and discharge samples, respectively. Notably, these genes showed significant isoform changes without differential gene expression (Suppl. Figure 2). Additionally, FECH and S100A9 comprise novel transcripts (MSTRG.27408.6, MSTRG.3657.4, and MSTRG.3657.8) that exhibit differential usage between focal and generalized seizures (Suppl. Figures 2a and c).

**Fig. 4:**
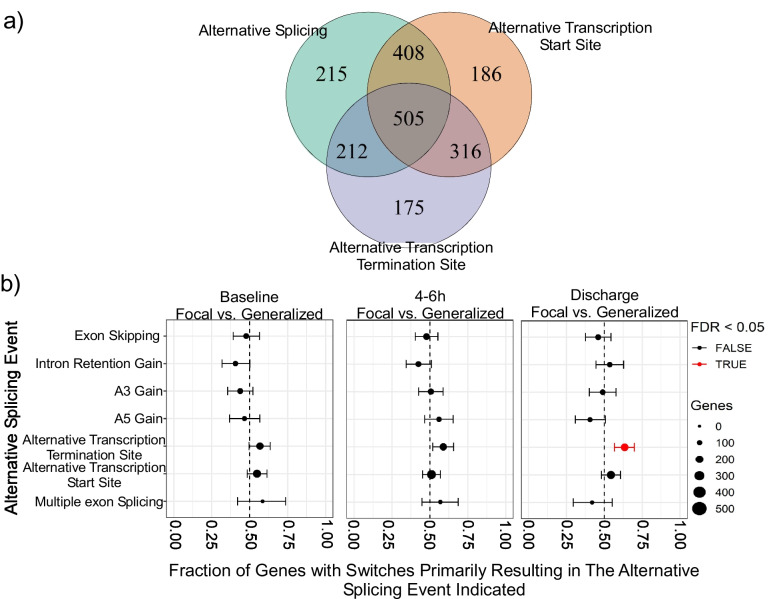
Mechanism and functional consequences of isoform switching in focal and generalized seizures **a** Isoform switching mechanisms involved alternative splicing (AS), alternative transcription starts sites (aTSS), and alternative transcription termination sites (aTTS). **b** Functional consequences of isoform switching includes alternative transcription starts sites (aTSS) gain as significant splicing event in discharge group

The consequence of these switching events on transcript function was then assed. We found a combination of alternative splicing (AS), alternative transcription starts sites (aTSS), and alternative transcription termination sites (aTTS) collectively influenced isoform switching. Specifically, 25.03% (505 genes) of isoform switching events were attributed to the interaction among these mechanisms (Fig. 4a). We noticed a significant (FDR-corrected *p* < 0.05) increase in alternative transcript termination site (aTTS) (Fig. 4b). These findings suggest that alterations in alternative splicing occur following focal and generalized seizure events.

### Gene Ontology of Differential Transcript Usage Following Seizures

**Fig. 5:**
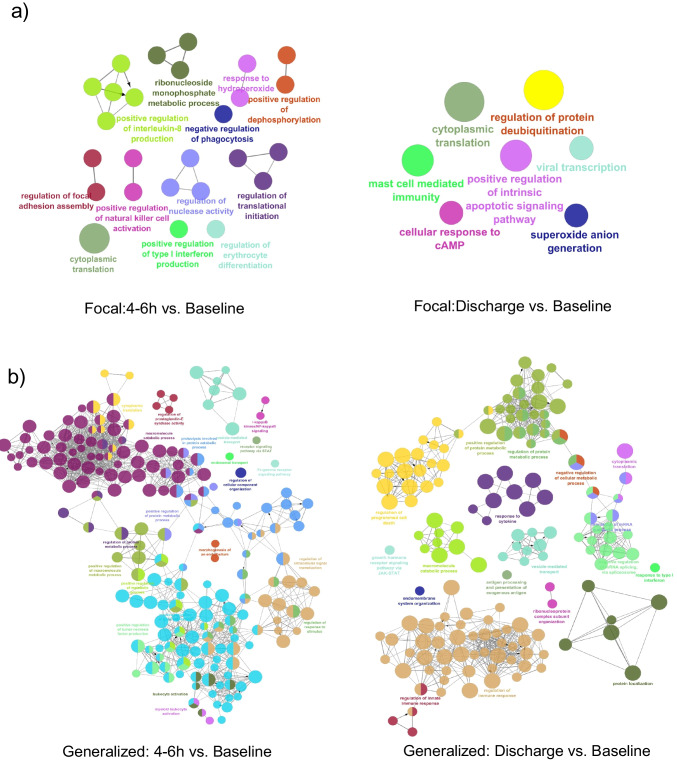
Gene ontology and pathway analysis of differential transcript usage genes following seizure types paired with baseline. Focal seizure: **a** At 4–6 h post-seizure, pathways related to acute inflammatory responses and dysregulation of cellular processes were prominent. **b** In the discharge group, enrichment shifts to protein ubiquitination and apoptotic pathways. Generalized seizure: **c** cellular activity and metabolic turnover were increased at 4–6 h post-seizure which **d** transitioning to immune response regulation by discharge. In contrast to focal seizures, generalized seizures show enrichment in distinct functional pathways, indicating a more coordinated molecular response

To understand the functional implications of genes undergoing switching events, we conducted gene ontology and pathway analyses. We performed functional enrichment analysis of differential transcript usage genes within the seizure groups. Focal seizure group enriched with acute inflammatory and dysregulation of cellular processes, especially protein synthesis and nucleotide metabolism. At discharge, these pathways shift to protein ubiquitination and apoptotic pathway (Fig. 5a and b). Conversely following generalized seizure, pathway analysis shows enhanced cellular activity and metabolic turnover which converge to immune response regulation at discharge (Fig. 5c and d). Notably, following focal seizures, the splicing events do not appear to converge around specific functional pathways. In contrast, following generalized seizures, we observe enrichment in distinct functional pathways.

We further observed significant enrichment across time points of focal and generalized seizure. In 4–6 h post-seizure group, significant activity is related to the regulation of mRNA splicing and ribonucleoprotein complex assembly (Suppl. Figure 3a) while in discharge group, the focus shifts to immune-related processes, such as lymphocyte activation, cytokine production, and regulation of the immune response (Suppl. Figure 3b). This implies that the immediate post-seizure period is characterized by intense cellular and molecular adjustments, while the later stage involves a significant immune system response.

### Differential Transcript Usage Analysis for Seizure Sub-Type

**Fig. 6:**
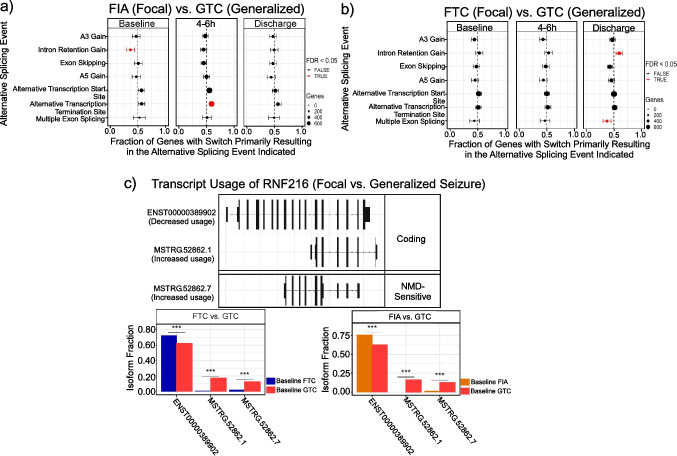
Identification of signatures associated with seizure sub-types across time points. **a** Statistically significant functional alterations are observed in comparisons including baseline FIA vs. GTC (intron retention gain), 4–6 h FIA vs. GTC (alternative transcription termination sites), and **b** discharge FTC vs. GTC (intron retention gain and multiple exon skipping). **c** RNF216 exhibits common isoform switching in baseline FIA and FTC compared to GTC. FIA, focal with impaired awareness; FTC, focal seizures time course; GTC, generalized tonic clonic]

We further refined signatures associated with sub-type of focal (FIA and FTC) and generalized seizure (GTC) across time points. We identified significant intron retention loss in FIA patients and alternative transcript termination site gain events in GTC patients at baseline and 4–6 h post-seizure, respectively (Fig. 6a**)**. However, intron retention gains with loss of multiple exons skipping events were noticed at discharge in GTC vs. FTC patients (Fig. 6b). We prioritized the top-ranked genes and identified BCL2 (baseline), EIF4G2 (4–6 h post-seizure), and TPI1 (discharge) on FIA vs. GTC comparison and S100A9 (baseline), RAB18 (4–6 h post-seizure), and RN7SL1 (discharge) on FTC vs. GTC comparison. Noticeably, RNF216 was particularly significant in baseline FIA and FTC compared to GTC, with notable NMD-sensitive isoforms (Fig. 6c). These preliminary data suggest patterns of isoform splicing are different following different sub-types of seizure.

### ML-Based Seizure Prediction Highlights the Difference Between Focal and Generalized Seizures

**Fig. 7:**
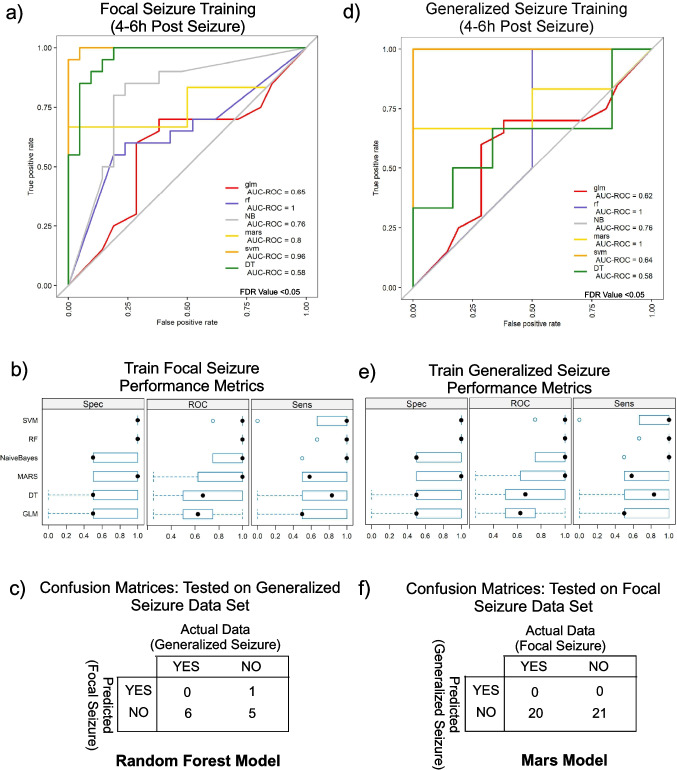
ML-based modeling to distinguish between focal and generalized seizure. **a** Classification models were trained using six algorithms on baseline versus 4–6 h post-seizure data from focal seizure patients. Random forest, Naïve Bayes, and radial SVM exhibited the highest performance with AUC-ROC values ranging from 0.95 to 1.0. These models achieved 100% accuracy on training data and correctly classified generalized seizure data without misclassification. **b** Box plots summarize the specificity, AUC-ROC, and sensitivity metrics for models trained on focal seizure data, showing the distribution of values across different models. **c** A confusion metric presents the classification results when the focal seizure-trained model was tested on the generalized seizure dataset, highlighting actual versus predicted classifications. **d** A second model was trained on generalized seizure data using the same algorithms, with MARS performing best, achieving an AUC-ROC value of 1.0. **e** Box plots depicting performance variation across different models while **f** confusion metrics summarized 100% accuracy, correctly identifying all focal seizure data as not generalized seizures. (model abbreviations: glm, generalized linear model; rf, random forest, NB, naïve Bayes; mars, multivariate adaptive regression splines, svm, support vector machine, DT, decision tree)

We investigated whether ML prediction modeling could be used to identify patients who have a specific seizure sub-type vs. a non-seizure (baseline) sample. The goal of the modeling was to determine whether a focal seizure would be identified as a generalized seizure and vice-versa. Classifier lists of transcripts were initially established following differential transcript expression analysis to identify baseline vs 4–6 h differential transcript expression following focal seizure (Fig. 1c). We first created a training dataset of focal seizure samples, consisting of baseline samples from patients with EEG-confirmed focal seizure (*N* = 21) and 4–6 h post-focal seizure samples (train = 20). The testing set consisted of baseline and 4–6 h samples from patients with EEG-verified generalized seizure (*N* = 6 of each). Using the training data (focal), we trained classifiers using six algorithms from the caret package, as depicted in Fig. 7a. The model was trained on baseline versus 4–6 h post-seizure data from patients who had experienced a focal seizure (21 patients; two samples each), with each subjected to ten-fold cross-validation. The AUC-ROC, sensitivity, and specificity of cross validation testing are given in Fig. 7a and b; random forest, Naïve Bayes, and radial SVM models exhibited the highest performance during training (mean AUC-ROC values ranging from 0.95 to 1.0) (Fig. 7a). The training model when applied to the training data achieved 100% accuracy as expected. These models were subsequently tested on baseline and 4–6 h generalized seizure data (six patients; two samples each). The models classified 11 samples as no focal seizure (N) (Fig. 7c). A second model was trained only the generalized seizure data using the same algorithms (generalized train). Based on AUC-ROC analysis, the multivariate adaptive regression splines (mars) model performed best, achieving an AUC-ROC value of 1.0 (Fig. 7d). The performance evaluation of these classifiers is shown in Fig. 7e. This model was then tested on the focal seizure data, and it classified all samples as no generalized seizure (N), correctly identifying focal seizure data as not generalized seizures (Fig. 7f). These data suggest ML models based on focal seizure data do not misclassify generalized seizure data and vice versa.

We also performed prediction modeling using differentially expressed transcripts between baseline and discharge samples following EEG-verified focal seizures (Fig. 1c). Using the same models and parameters as for the 4–6 h data, the random forest algorithm showed good training performance on the focal seizure dataset. However, the model misclassified one baseline sample as a focal seizure and two generalized seizures as focal seizures (Suppl. Figure 4a). Similarly, training on the generalized seizure dataset yielded acceptable training performance (RF accuracy = 0.9), but the model struggled to correctly classify generalized seizures, misclassifying seven focal seizures and 13 baseline samples as generalized seizures (Suppl. Figure 4b). These findings suggest that the 4–6 h data may be more predictive, or that the model requires a larger dataset for improved classification.

## Discussion

We investigated transcript expression and splicing variants in response to focal and generalized epileptic seizures, aiming to understand their potential to discriminate between seizure types. We generated RNA-Seq dataset across three time points (baseline, 4–6 h post-seizure, at discharge) and identified novel transcripts. Specifically, 13.48% of these transcripts mapped entirely to unannotated regions, and 5.33% mapped partially to annotated regions. Generalized seizures exhibited more pronounced transcriptomic changes and alternative splicing than focal seizures, even at baseline, with no overlap in transcriptomic responses between the two. Significant differences in transcript expression were observed at each time point, with greater changes occurring 4–6 h post-seizure and at discharge in generalized seizures compared to focal seizures. Sub-type analysis revealed differences between focal to bilateral tonic–clonic (FTC) and generalized tonic–clonic (GTC) seizures, as well as between focal impaired awareness (FIA) and GTC seizures, but not between FIA and FTC seizures. Machine learning models effectively distinguished focal from generalized seizures without misclassification, highlighting blood RNA profiles as a potential tool for seizure classification and therapeutic decision-making.

Recent research has highlighted the significance of blood in detecting physiological and pathological events within the brain [11, 22]. The brain’s lymphatic system harbors lymphocytes, which are susceptible to environmental stress resulting from neurological insults [23–26]. Seizures can induce changes in brain endothelial cells, potentially compromising the blood–brain barrier integrity, facilitating the diffusion of factors or immune cells, and leading to transcriptome alterations [27]. Together these data suggest blood transcriptome analysis, especially differential transcript usage may enable the retrospective diagnosis of seizure types.

Our findings align with previous research emphasizing the importance of exploring unannotated transcriptome regions that may enhance our understanding of epilepsy and potentially identify biomarkers or therapeutic targets [28–31]. At the outset, we define “biomarkers” in this study as surrogate biomarkers, which are indicators that, while not necessarily establishing a mechanistic link to the disease, can be useful for clinical diagnostics. This definition is consistent with the NIH guidelines, which also recognize surrogate biomarkers as valid for disease [32].

Generalized seizures induced a more profound and persistent transcriptional response, with a larger number of differentially expressed transcripts at both 4–6 h post-seizure and discharge. In contrast, focal seizures exhibited fewer differentially expressed transcripts but showed distinct temporal dynamics, with specific transcripts consistently dysregulated across time points. Isoform switching events were prevalent in both seizure types, but generalized seizures showed a higher number of unique switching events, particularly at 4–6 h post-seizure and discharge. Functional pathway enrichment analysis revealed that generalized seizures were characterized by immediate cellular and molecular adjustments, followed by a significant immune response at discharge. While focal seizures had less convergence around specific pathways, indicating a more varied transcriptomic response. This is consistent with previous studies showing altered expression of transcriptome in brain following various disorders and highlighting its role between healthy and diseased brain [33–35].

To the best of our knowledge, there are few NGS gene expression studies in blood following seizure, and no prior investigation has delved into isoform switching for retrospective seizure type diagnosis [36–38]. Our comparative analyses of patients with seizure types as well as sub-types revealed several genes having significant isoform switching that have never been associated with seizure. Interestingly, many of them were not detected by conventional gene and transcript expression analysis. Another interesting finding was that isoform switching showed substantial heterogeneity between seizure types across different time points. We also observed a substantial number of switches occur between protein coding and noncoding transcript isoforms, thereby affecting the overall protein level. Many of these isoform switches were predicted to have functional consequences such as intron retention gain and alternative transcription termination sites. Gene ontology analysis uncovers mRNA splicing, immune signaling, and acute inflammatory pathways following seizures that also have been shown previously [39–41].

We identified RNF216 as inducing the expression of its novel NMD-sensitive transcript (MSTRG.52862.7) across following generalized seizure. RNF216 (E3 ubiquitin ligase) is involved in ubiquitin–proteasome system, and its mutations are associated with a rare neurodegenerative disorder known as Gordon Holmes syndrome [42, 43]. Its consistent presence in both the comparisons between focal (FTC and FIA) vs. generalized (GTC) seizure types revealed unique post-transcriptional regulatory mechanisms associated with different seizure types. RNF216 involvement following generalized seizures may be linked to its functions in protein ubiquitination and degradation pathways, which are crucial for maintaining cellular homeostasis and regulating immune responses. The NMD sensitivity of MSTRG.52862.7 also highlights its potential role as a biomarker for differentiating between seizure types. These data show potential exciting mechanisms of how seizure affects various genes and cellular systems. However, we cannot infer what such changes may mean for the brain, seizure mechanisms, or epileptogenesis. The splicing events we describe are in the sequalae following the seizure.

We investigated the ability of RNA transcript profiles to distinguish between seizure types, using prediction ML modeling in the caret package. We investigated 6 ML models and found random forest to perform best with focal seizures training and generalized seizure testing. We only had six generalized seizure samples which may explain the poorer performance in training of this data set. Our goal was to determine whether a patient has had a focal seizure vs. no seizure or a generalized seizure vs. no seizure. For this, we used baseline samples as a control. In future studies, a collection of other conditions with similar clinical presentation as a seizure (seizure mimic) will be necessary to determine the utility of this approach. Given some seizure types show better response to anti-seizure medications than others, this approach could be used in the initial triage of a seizure patient and potentially help identify effective anti-seizure therapies.

In summary, we explored transcriptomic alterations and splicing variants in response to epileptic seizures, aiming to identify biomarkers for distinguishing seizure types. We utilized a comprehensive RNA-Seq dataset across multiple time points and discovered numerous novel transcripts, particularly in unannotated regions of the genome, underscoring the importance of exploring these areas for potential biomarkers. Our findings reveal distinct transcriptomic profiles in focal versus generalized seizures, with significant isoform switching events that have not been previously linked to seizures. These data show the potential of point of care genomics to retrospectively distinguish seizure types after the seizure event.

## Limitation

We acknowledge limitations including modest sample size (being performed throughout the COVID pandemic and limited by sponsor funding). Furthermore, longitudinal analyses may be required to capture the detailed temporal dynamics of RNA changes following a spell. In our study, we investigated up to 24 h post-seizure, but it is unclear what the temporal extinction profile of such RNA changes may be. Our plans are to expand this study to a larger cohort of patients over additional time points post-seizure. The role of isoform switches with respect to brain seizure is unclear. Future studies could employ CRISPR-mediated isoform modulation in neuronal models to target these switches to assess downstream role or impact of such blood-based gene changes on epilepsy or seizure activity. Serial assessment of isoform abundance aids in mitigating confounding factors, although obtaining time-series samples poses challenges. We lack the ability to control the timing of seizure, number of seizures, anti-seizure medications administered, rescue medication given or discharge timing. Additional sample sizes may enable such subgroup analyses. Further, such analyses may help identify patients who are refractory to various anti-seizure medications. Our modeling suggest blood RNA profiles can distinguish between focal and generalized seizure types, but we lacked an independent validation set for both data sets. Our plans include validation of our findings using an independent cohort to assess generalizability. Further, functional validation of these findings will be imperative for clinical translation and assessment of utility. Many other neurological conditions may be presented as a seizure or temporary loss of consciousness; hence, studies including the likely differential diagnosis of a seizure will be necessary.

## Supplementary Information

Below is the link to the electronic supplementary material.

Supplementary file1 (DOCX 36 KB)

Supplementary file2 (PDF 3078 KB)

## Data Availability

Sequencing data and clinical phenotype data are available at dbGAP (study phs003460.v1.p1). All data generated or analyzed during this study are included in this published article and its supplementary information files. Raw expression data and copies of scripts are available at (https://github.com/rob-meller or https://github.com/Vermarashi/).

## References

[1] Anwar H et al (2020) Epileptic seizures. Discoveries (Craiova) 8(2):e110 32577498 10.15190/d.2020.7 PMC7305811

[2] Scheepers B, Clough P, Pickles C (1998) The misdiagnosis of epilepsy: findings of a population study. Seizure 7(5):403–406 9808117 10.1016/s1059-1311(05)80010-x

[3] Ulate-Campos A et al (2016) Automated seizure detection systems and their effectiveness for each type of seizure. Seizure 40:88–101 27376911 10.1016/j.seizure.2016.06.008

[4] Maiti R et al (2018) Effect of anti-seizure drugs on serum S100B in patients with focal seizure: a randomized controlled trial. J Neurol 265(11):2594–2601 30173303 10.1007/s00415-018-9026-1

[5] Chen DK et al (2005) Use of serum prolactin in diagnosing epileptic seizures: report of the Therapeutics and Technology Assessment Subcommittee of the American Academy of Neurology. Neurology 65(5):668–675 16157897 10.1212/01.wnl.0000178391.96957.d0

[6] Maiti R et al (2017) Effect of carbamazepine and oxcarbazepine on serum neuron-specific enolase in focal seizures: a randomized controlled trial. Epilepsy Res 138:5–10 29028517 10.1016/j.eplepsyres.2017.10.003

[7] Kwan P, Schachter SC, Brodie MJ (2011) Drug-resistant epilepsy. N Engl J Med 365(10):919–926 21899452 10.1056/NEJMra1004418

[8] Bullinger K et al (2025) Retrospective discrimination of PNES and epileptic seizure types using blood RNA signatures. J Neurol 272:128. 10.1007/s00415-024-12877-1 10.1007/s00415-024-12877-1 PMC11735489 39812831

[9] Jickling GC et al (2010) Signatures of cardioembolic and large-vessel ischemic stroke. Ann Neurol 68(5):681–692 21031583 10.1002/ana.22187 PMC2967466

[10] Jickling GC et al (2013) RNA in blood is altered prior to hemorrhagic transformation in ischemic stroke. Ann Neurol 74(2):232–240 23468366 10.1002/ana.23883 PMC3752310

[11] Meller R et al (2016) Blood transcriptome changes after stroke in an African American population. Ann Clin Transl Neurol 3(2):70–81 26900583 10.1002/acn3.272 PMC4748310

[12] Fisher RS et al (2017) Operational classification of seizure types by the International League Against Epilepsy: position paper of the ILAE Commission for Classification and Terminology. Epilepsia 58(4):522–530 28276060 10.1111/epi.13670

[13] Langmead B, Salzberg SL (2012) Fast gapped-read alignment with Bowtie 2. Nat Methods 9(4):357–359 22388286 10.1038/nmeth.1923 PMC3322381

[14] Dobin A et al (2013) STAR: ultrafast universal RNA-seq aligner. Bioinformatics 29(1):15–21 23104886 10.1093/bioinformatics/bts635 PMC3530905

[15] Kovaka S et al (2019) Transcriptome assembly from long-read RNA-seq alignments with StringTie2. Genome Biol 20(1):278 31842956 10.1186/s13059-019-1910-1 PMC6912988

[16] Robinson MD, McCarthy DJ, Smyth GK (2010) edgeR: a bioconductor package for differential expression analysis of digital gene expression data. Bioinformatics 26(1):139–140 19910308 10.1093/bioinformatics/btp616 PMC2796818

[17] Ritchie ME et al (2015) limma powers differential expression analyses for RNA-sequencing and microarray studies. Nucleic Acids Res 43(7):e47 25605792 10.1093/nar/gkv007 PMC4402510

[18] Vitting-Seerup K, Sandelin A (2017) The landscape of isoform switches in human cancers. Mol Cancer Res 15(9):1206–1220 28584021 10.1158/1541-7786.MCR-16-0459

[19] Shannon P et al (2003) Cytoscape: a software environment for integrated models of biomolecular interaction networks. Genome Res 13(11):2498–2504 14597658 10.1101/gr.1239303 PMC403769

[20] Bindea G et al (2009) ClueGO: a cytoscape plug-in to decipher functionally grouped gene ontology and pathway annotation networks. Bioinformatics 25(8):1091–1093 19237447 10.1093/bioinformatics/btp101 PMC2666812

[21] Kuhn M (2008) Building predictive models in R using the caret Package. J Stat Softw 28(5):1–2627774042

[22] Hardy JJ et al (2017) Assessing the accuracy of blood RNA profiles to identify patients with post-concussion syndrome: a pilot study in a military patient population. PLoS ONE 12(9):e0183113 28863142 10.1371/journal.pone.0183113 PMC5581162

[23] Sharp FR et al (2011) Molecular markers and mechanisms of stroke: RNA studies of blood in animals and humans. J Cereb Blood Flow Metab 31(7):1513–1531 21505474 10.1038/jcbfm.2011.45 PMC3137473

[24] Sharp FR et al (2007) Genomic profiles of stroke in blood. Stroke 38(2 Suppl):691–693 17261717 10.1161/01.STR.0000247940.27518.38

[25] Jickling GC et al (2012) Prediction of cardioembolic, arterial, and lacunar causes of cryptogenic stroke by gene expression and infarct location. Stroke 43(8):2036–2041 22627989 10.1161/STROKEAHA.111.648725 PMC3422649

[26] Stamova B et al (2010) Gene expression profiling of blood for the prediction of ischemic stroke. Stroke 41(10):2171–2177 20798371 10.1161/STROKEAHA.110.588335 PMC2987675

[27] Louveau A et al (2015) Structural and functional features of central nervous system lymphatic vessels. Nature 523(7560):337–341 26030524 10.1038/nature14432 PMC4506234

[28] Qureshi IA, Mehler MF (2012) Emerging roles of non-coding RNAs in brain evolution, development, plasticity and disease. Nat Rev Neurosci 13(8):528–541 22814587 10.1038/nrn3234 PMC3478095

[29] Kadakkuzha BM, Puthanveettil SV (2013) Genomics and proteomics in solving brain complexity. Mol Biosyst 9(7):1807–1821 23615871 10.1039/c3mb25391k PMC6425491

[30] Gandal MJ et al (2018) Shared molecular neuropathology across major psychiatric disorders parallels polygenic overlap. Science 359(6376):693–697 29439242 10.1126/science.aad6469 PMC5898828

[31] Yazarlou F, Lipovich L, Loeb JA (2024) Emerging roles of long non-coding RNAs in human epilepsy. Epilepsia 65(6):1491–1511 38687769 10.1111/epi.17937 PMC11166529

[32] Katz R (2004) Biomarkers and surrogate markers: an FDA perspective. NeuroRx 1(2):189–195 15717019 10.1602/neurorx.1.2.189 PMC534924

[33] Kobow K, El-Osta A, Blumcke I (2013) The methylation hypothesis of pharmacoresistance in epilepsy. Epilepsia 54(Suppl 2):41–47 23646970 10.1111/epi.12183

[34] Arion D et al (2017) Transcriptome alterations in prefrontal pyramidal cells distinguish schizophrenia from bipolar and major depressive disorders. Biol Psychiatry 82(8):594–600 28476208 10.1016/j.biopsych.2017.03.018 PMC5610065

[35] Munji RN et al (2019) Profiling the mouse brain endothelial transcriptome in health and disease models reveals a core blood-brain barrier dysfunction module. Nat Neurosci 22(11):1892–1902 31611708 10.1038/s41593-019-0497-x PMC6858546

[36] Loscher W (2002) Animal models of epilepsy for the development of antiepileptogenic and disease-modifying drugs. A comparison of the pharmacology of kindling and post-status epilepticus models of temporal lobe epilepsy. Epilepsy Res 50(1–2):105–23 12151122 10.1016/s0920-1211(02)00073-6

[37] Henshall DC, Simon RP (2005) Epilepsy and apoptosis pathways. J Cereb Blood Flow Metab 25(12):1557–1572 15889042 10.1038/sj.jcbfm.9600149

[38] Jamali S et al (2006) Large-scale expression study of human mesial temporal lobe epilepsy: evidence for dysregulation of the neurotransmission and complement systems in the entorhinal cortex. Brain 129(Pt 3):625–641 16399808 10.1093/brain/awl001

[39] Thompson SJ et al (2011) Suppression of TNF receptor-1 signaling in an in vitro model of epileptic tolerance. Int J Physiol Pathophysiol Pharmacol 3(2):120–132 21760970 PMC3134006

[40] Li G et al (2011) Cytokines and epilepsy. Seizure 20(3):249–256 21216630 10.1016/j.seizure.2010.12.005

[41] Ahl M et al (2023) Immune response in blood before and after epileptic and psychogenic non-epileptic seizures. Heliyon 9(3):e13938 36895367 10.1016/j.heliyon.2023.e13938 PMC9988551

[42] Husain N et al (2017) TRIAD3/RNF216 mutations associated with Gordon Holmes syndrome lead to synaptic and cognitive impairments via Arc misregulation. Aging Cell 16(2):281–292 27995769 10.1111/acel.12551 PMC5334534

[43] Margolin DH et al (2013) Ataxia, dementia, and hypogonadotropism caused by disordered ubiquitination. N Engl J Med 368(21):1992–2003 23656588 10.1056/NEJMoa1215993 PMC3738065

