# Supplementary material for: Differential Transcript Expression and Alternative RNA Splicing Patterns to Differentiate Focal vs. Generalized-Onset Seizures
Source: Mol Neurobiol. 2025 Jun 11;62(10):13303–17. doi: 10.1007/s12035-025-05110-1 (PMC12433373; doi:10.1007/s12035-025-05110-1)
Supplement: Supplementary file 1 — Supplementary file1 (DOCX 36 KB) [file 12035_2025_5110_MOESM1_ESM.docx]

**Supplementary Data**

**Suppl. Table 1: corrected p-values from Training models.**

Focal Trained Model:

|  | FocalTrainedModel | P_value | P_value_Bonferroni | P_value_FDR |
| --- | --- | --- | --- | --- |
| 1 | MARS | 1.22x10^-12^ | 7.33x10^-12^ | 1.47x10^-12^ |
| 2 | GLM | 1.22x10^-12^ | 7.33x10^-12^ | 1.47x10^-12^ |
| 3 | RF | 1.22x10^-12^ | 7.33x10^-12^ | 1.47x10^-12^ |
| 4 | SVM | 1.22x10^-12^ | 7.33x10^-12^ | 1.47x10^-12^ |
| 5 | NB | 1.22x10^-12^ | 7.33x10^-12^ | 1.47x10^-12^ |
| 6 | DT | 4.93x10^-06^ | 2.96x10^-05^ | 4.93x10^-06^ |

Generalized Trained Model:

|  | GeneralizedTrainedModel | P_value | P_value_Bonferroni | P_value_FDR |
| --- | --- | --- | --- | --- |
| 1 | MARS | 0.0031740 | 0.0190440 | 0.0038088 |
| 2 | GLM | 0.0002441 | 0.0014646 | 0.00036615 |
| 3 | RF | 0.0002441 | 0.0014646 | 0.00036615 |
| 4 | SVM | 0.0002441 | 0.0014646 | 0.00036615 |
| 5 | NB | 0.0002441 | 0.0014646 | 0.00036615 |
| 6 | DT | 0.0412300 | 0.2473800 | 0.04123 |

**Script**

#### Prediction Modeling: Seizure project

## Edited by RMELLER

## REFS https://github.com/rob-meller/SeizurePaper_1/blob/main/Lasso_SEIZURE.MS.FINAL.R

## REFS https://topepo.github.io/caret/index.html

## REFS https://www.machinelearningplus.com/machine-learning/caret-package/

# Intital steps

setwd("C:/Users/rmeller/Desktop/LASSO_SEIZURE/RV-DATA")

getwd()

# Data are in a folder called "DATA"

# Create a folder called "PLOTS" for results

library(readr)

library(corrplot)

library(caret)

library(glmnet)

library(stringr)

library(plyr)

library(dplyr)

library(tidyr)

library(skimr)

library(caretEnsemble)

library(reshape2)

library(pROC)

library(caretSDM)

library(rminer)

library(MLeval)

##Load Datasheet (TMM gene expression values calculated in Partek Genomics Studio)

df_0 <- read.table("SEIZURE_GENE_TMM.txt", sep = "\t", header = TRUE); typeofdata="Gene tmm"

df_0[1:20,1:20]

#Remove S0994 unknown etiology

df1<- df_0[,1:19]

df1 <- df1[!df1$Sample=="S994",]

df1[1:10,1:19];dim(df1)# 89 samples

# Create test and training data frames

## SEIZ samples

df2 = df1[(df1$"EEG.change"=="YES"),]

df2[1:10,1:19];dim(df2)# 78 samples

# need to get sample IDs to match. Split df1

df_2 <- df2 %>% separate("Sample.ID", c("SAMPLE", "JUNK"), sep = "_Co")

df_2$JUNK <- NULL

# save here

write.table(df_2, "SeizurePhenodata.txt", sep="\t")

# quick clean up in excel to edit sampleIDs and remove a few columns

df_pheno <- read.table("SeizurePhenodata.txt", sep = "\t", header = TRUE); typeofdata="Phenotype"

dim(df_pheno)

head(df_pheno)

colnames(df_pheno)[1] <- ("SAMPLES")

## load in Transcript count matrix

df_cpm <- read.table("cpm_matrix.csv", sep = ",", header = TRUE); typeofdata="transcript counts"

dim(df_cpm)

rownames(df_cpm) <- df_cpm$X

df_cpm$X <- NULL

dim(df_cpm)

# first transpose cpm matrix

df_cpm1 <- data.frame(t(df_cpm))

df_cpm1$SAMPLES<- rownames(df_cpm1)

df_cpm1[1:10,1:10]

dim(df_pheno); dim(df_cpm1)

df_merge <- merge(df_pheno,df_cpm1, by="SAMPLES")

df_merge[1:10,1:10]

dim(df_merge);# 77 x 299241 better!!

## remove discharge

df3 = df_merge[!df_merge$Time=="Disc",]

df3[1:10,1:17];dim(df3)# 53 samples

# Split Focal and generalized

df4 <-df3[(df3$"Type.of.Seizure"=="Generalized"),]

df3 <-df3[(df3$"Type.of.Seizure"=="Focal"),]

df3[1:10,1:5]

# To address error message, convert df to data frame object

train_df <- as.data.frame(df3)

test_df <- data.frame(df4[,])

train_df <- train_df[,c(3,18:299241)]

train_df[1:10,1:20]; dim(train_df)

##Pull in data sheet of sig genes..

genes=read.csv("4_6h_Focal.csv")

genes[1:20,1:5]; dim(genes) # 60x26 # Genes are in colum Gene.Symbol

## Now subset gene expression values based on list of sig genes.

train_df_0 <- (train_df[,names(train_df) %in% genes$"X"])

head(train_df_0);dim(train_df_0)

# now add back Time info

dfz=cbind(train_df$Time, train_df_0[,])

# rename col1

colnames(dfz)[1] <- "Time"

head(dfz); dim(dfz)

## rename back to train_df

train_df = dfz

## Data needs to be a binomial/ classifier model!!! recode data to Y/ N

train_df$Time <- recode(train_df$Time, 'Base' = 'N', '4-6h'='Y',

.default=NA_character_)

head(train_df); dim(train_df)

# better recode the test data

test_df$Time <- recode(test_df$Time, 'Base' = 'N', '4-6h'='Y',

.default=NA_character_)

head(test_df); dim(test_df)

# quick assessment..

skim(train_df)

#############################################################################

# PREDICTION MODELING #

#############################################################################

##Preprocessing

preProcess_range_model <- preProcess(train_df, method=c('scale', 'center'))

train_df <- predict(preProcess_range_model, newdata = train_df)

# process the test data too

test_df <- predict(preProcess_range_model, newdata = test_df)

# Review expression of DEGs

featurePlot(x = train_df[, 2:42],

y = as.factor(train_df$Time),

plot = "box",

strip=strip.custom(par.strip.text=list(cex=.7)),

scales = list(x = list(relation="free"),

y = list(relation="free")))

featurePlot(x = train_df[, 2:42],

y = as.factor(train_df$Time),

plot = "density",

strip=strip.custom(par.strip.text=list(cex=.7)),

scales = list(x = list(relation="free"),

y = list(relation="free")))

## Let's try a series of models...

# Define the training control

fitControl <- trainControl(

method = 'cv', # k-fold cross validation

number = 10, # number of folds

savePredictions = 'final', # saves predictions for optimal tuning parameter

classProbs = TRUE, # should class probabilities be returned

summaryFunction=twoClassSummary # results summary function

)

# Train the model using earth

set.seed(1234); model_mars = train(Time~ .,

data=train_df, method='earth', tuneLength=20, trControl = fitControl)

varimp_mars <- varImp(model_mars)

res_mars <- evalm(model_mars)

# Train the model using glm

set.seed(1234); model_glm = train(Time~ .,

data=train_df, method='glm', tuneLength=20, trControl = fitControl)

varimp_glm <- varImp(model_glm)

res_glm <- evalm(model_glm)

# Train the model using rf

set.seed(1234); model_rf = train(Time~ .,

data=train_df, method='rf', tuneLength=20, trControl = fitControl, importance=TRUE)

varimp_rf <- varImp(model_rf)

res_rf <- evalm(model_rf)

# Train the model using svm

set.seed(1234); model_svmRadial = train(Time~ .,

data=train_df, method='svmRadial', tuneLength=10, trControl = fitControl, importance=TRUE)

svm.imp <-Importance(model_svmRadial, data=train)

res_SVM <- evalm(model_svmRadial)

# Train the model using naive Bayes

set.seed(1234); model_NB = train(Time~ .,

data=train_df, method='naive_bayes', tuneLength=10, trControl = fitControl)

varimp_NB <- varImp(model_NB)

res_NB <- evalm(model_NB)

# Train the model using Gradient Boost

set.seed(1234); model_GBM = train(Time~ .,

data=train_df, method='gbm', tuneLength=10, trControl = fitControl)

varimp_GBM <- summary.gbm(model_GBM)

res_GBM <- evalm(model_GBM)

# Train the model using decision trees

set.seed(1234); model_DT = train(Time~ .,

data=train_df, method='rpart', tuneLength=10, trControl = fitControl)

varimp_DT <- varImp(model_DT)

res_DT <- evalm(model_DT)

## Then compare model performances using resample()

models_compare <- resamples(list(GLM=model_glm, RF=model_rf,

NaiveBayes=model_NB,MARS=model_mars,

SVM=model_svmRadial, DT=model_DT))

# Summary of the models performances

sink("1.SEIZURE_Training set.txt")

cat("Summary of Training using ROC metrics")

print(summary(models_compare))

sink()

# Plot ROCs

test4 <- evalm(list(GLM=model_glm, RF=model_rf,

NaiveBayes=model_NB,MARS=model_mars,

SVM=model_svmRadial, DT=model_DT),

gnames=c('glm', 'rf', 'NB', 'mars', 'svm', 'DT'),rlinethick=0.8,fsize=8,

plots='r')

tiff(file="PLOTS/1.Seizure_train__ROC.tiff", unit= "in", res = 300, width = 6, height = 6)

test4$roc

dev.off()

# Draw box plots to compare models

tiff(file="PLOTS/1.Seizure_train_Box_comp).tiff", unit= "in", res = 300, width = 6, height = 6)

scales <- list(x=list(relation="free"), y=list(relation="free"))

bwplot(models_compare, scales=scales)

dev.off()

#############################################################################

# TESTING #

#############################################################################

# Predict on test. Data print to summary sheet

sink("2.SEIZURE model testing.txt")

cat("Summary of Testing predictions using confusion matrix.\n")

cat("Reference testing population = 6 baseline GEN, 6 post GEN pateints.\n")

cat("Reference training population = 21 baseline Focal, 20 post FOCAL pateints.\n")

# model_mars

cat("MARS-model on test\n")

predicted_test <- predict(model_mars, newdata=test_df)

cat("Predicted values\n")

print(predicted_test)

print(confusionMatrix(reference = as.factor(test_df$Time), data = predicted_test,

mode='everything', positive='Y'))

cat("MARS-model on train\n")

#train

predicted_train=predict(model_mars, newdata=train_df)

print(predicted_train)

print(confusionMatrix(reference = as.factor(train_df$Time),

data = predicted_train, mode='everything', positive='Y'))

#model_glm

cat("glm model on test\n")

predicted_test <- predict(model_glm, newdata=test_df)

print(predicted_test)

print(confusionMatrix(reference = as.factor(test_df$Time), data = predicted_test,

mode='everything', positive='Y'))

#train

cat("glm model on train\n")

predicted_train=predict(model_glm, newdata=train_df)

print(predicted_train)

print(confusionMatrix(reference = as.factor(train_df$Time), data = predicted_train,

mode='everything', positive='Y'))

#model_rf

cat("rf-model on test\n")

predicted_test <- predict(model_rf, newdata=test_df)

print(predicted_test)

print(confusionMatrix(reference = as.factor(test_df$Time), data = predicted_test,

mode='everything', positive='Y'))

#train

cat("rf model on train\n")

predicted_train=predict(model_rf, newdata=train_df)

print(predicted_train)

print(confusionMatrix(reference = as.factor(train_df$Time), data = predicted_train,

mode='everything', positive='Y'))

#model_NB

cat("Naive-Bayes model on test\n")

predicted_test <- predict(model_NB, newdata=test_df)

print(predicted_test)

print(confusionMatrix(reference = as.factor(test_df$Time), data = predicted_test,

mode='everything', positive='Y'))

#train

cat("Naive-Bayes model on train\n")

predicted_train=predict(model_NB, newdata=train_df)

print(predicted_train)

print(confusionMatrix(reference = as.factor(train_df$Time), data = predicted_train,

mode='everything', positive='Y'))

#model_GBM

cat("GBM model on test\n")

predicted_test <- predict(model_GBM, newdata=test_df)

print(predicted_test)

print(confusionMatrix(reference = as.factor(test_df$Time), data = predicted_test,

mode='everything', positive='Y'))

#train

cat("GBM model on train\n")

print(predicted_train)

print(confusionMatrix(reference = as.factor(train_df$Time), data = predicted_train,

mode='everything', positive='Y'))

#model_svmRadial

cat("svmRadial model on test\n")

predicted_test <- predict(model_svmRadial, newdata=test_df)

print(predicted_test)

print(confusionMatrix(reference = as.factor(test_df$Time), data = predicted_test,

mode='everything', positive='Y'))

#train

cat("svmRadial model on train\n")

predicted_train=predict(model_svmRadial, newdata=train_df)

print(predicted_train)

print(confusionMatrix(reference = as.factor(train_df$Time), data = predicted_train,

mode='everything', positive='Y'))

#model_DT

cat("Decision Tree model on test\n")

predicted_test <- predict(model_DT, newdata=test_df)

print(predicted_test)

print(confusionMatrix(reference = as.factor(test_df$Time), data = predicted_test,

mode='everything', positive='Y'))

#train

cat("Decision Tree model on train\n")

predicted_train=predict(model_DT, newdata=train_df)

print(predicted_train)

print(confusionMatrix(reference = as.factor(train_df$Time), data = predicted_train,

mode='everything', positive='Y'))

sink()

## Multiple using caretEnsembl.

# recall control parameters from above

trainControl <- trainControl(method='repeatedcv',

number=10,

repeats=3,

savePredictions=TRUE,

classProbs=TRUE)

algorithmList <- c('rf', 'earth','svmRadial','naive_bayes', 'rpart')

# Then run

set.seed(1234); models <- caretList(Time~.,

data=train_df,

trControl=trainControl,

methodList=algorithmList)

results <- resamples(models)

summary(results)

# plot results as a correlation

xyplot(resamples(models))

data <- modelCor(resamples(models))

data

# creating correlation matrix

library (corrplot)

corrplot(data, method = 'color', order = 'alphabet')

# prefer this one!

# Box plots to compare models

scales <- list(x=list(relation="free"), y=list(relation="free"))

bwplot(results, scales=scales)

# Combine the predictions of multiple models to form a final prediction

# Create the trainControl

stackControl <- trainControl(method="repeatedcv",

number=10,

repeats=3,

savePredictions=TRUE,

classProbs=TRUE)

algorithmList2 <- c('rf', 'svmRadial')

# Then run

set.seed(1234); teststack <- caretList(Time~.,

data=train_df,

trControl=stackControl,

methodList=algorithmList2)

results <- resamples(teststack)

summary(results)

## Combining models into single glm

set.seed(1234);stack.glm <- caretStack(teststack, method="glm", metric="ROC", trControl=stackControl)

print(stack.glm)

# Predict on testData

#test_df <- predict(preProcess_range_model, newdata = test_df)

predicted_test <- predict(stack.glm, newdata=test_df)

confusionMatrix(reference = as.factor(test_df$Time), data = predicted_test, mode='everything', positive='Y')

predicted_train=predict(stack.glm, newdata=train_df)

confusionMatrix(reference = as.factor(train_df$Time), data = predicted_train, mode='everything', positive='Y')

#############################################################################

#############################################################################

###Now create a model on the Generalized data

# Create test and training data frame

# To address error message, convert df to data frame object

trainG_df <- as.data.frame(df4)

testG_df <- data.frame(df3[,])

dim(trainG_df)

trainG_df <- trainG_df[,c(3,18:299241)]

trainG_df[1:10,1:20]; dim(train_df)

##Pull in data sheet of sig genes..

genes=read.csv("4_6h_Gen.csv")

genes[1:20,1:5]; dim(genes) # 74x27 # Genes are in colum Gene.Symbol

## Now subset gene expression values based on inclusion in list.

train_df_0 <- (trainG_df[,names(trainG_df) %in% genes$"X"])

head(train_df_0);dim(train_df_0)

# now add back Time info

dfz=cbind(trainG_df$Time, train_df_0[,])

# rename col1

colnames(dfz)[1] <- "Time"

head(dfz); dim(dfz)

## rename back to train_df

train_df = dfz

## Data needs to be a binomial/ classifier model!!! recode data to A and B or Y/ N

train_df$Time <- recode(train_df$Time, 'Base' = 'N', '4-6h'='Y',

.default=NA_character_)

head(train_df); dim(train_df)

# better recode the test data

testG_df$Time <- recode(testG_df$Time, 'Base' = 'N', '4-6h'='Y',

.default=NA_character_)

head(testG_df); dim(testG_df)

# quick assessment..

skim(train_df)

#############################################################################

# PREDICTION MODELING #

#############################################################################

##Preprocessing

preProcess_range_model <- preProcess(train_df, method=c('scale', 'center'))

train_df <- predict(preProcess_range_model, newdata = train_df)

# process the test data too

test_df <- predict(preProcess_range_model, newdata = test_df)

#preProcess_range_model <- preProcess(train_df, method='pca')

#train_df <- predict(preProcess_range_model, newdata = train_df)

dim(train_df)

featurePlot(x = train_df[, 2:51],

y = as.factor(train_df$Time),

plot = "box",

strip=strip.custom(par.strip.text=list(cex=.7)),

scales = list(x = list(relation="free"),

y = list(relation="free")))

featurePlot(x = train_df[, 2:51],

y = as.factor(train_df$Time),

plot = "density",

strip=strip.custom(par.strip.text=list(cex=.7)),

scales = list(x = list(relation="free"),

y = list(relation="free")))

## Lets try all of these together...

set.seed(1234)

# Define the training control

fitControl <- trainControl(

method = 'cv', # k-fold cross validation

number = 10, # number of folds

savePredictions = 'final', # saves predictions for optimal tuning parameter

classProbs = TRUE, # should class probabilities be returned

summaryFunction=twoClassSummary # results summary function

)

# Train the model using earth

set.seed(1234); model_mars = train(Time~ .,

data=train_df, method='earth', tuneLength=20, trControl = fitControl)

# Train the model using glm

set.seed(1234); model_glm = train(Time~ .,

data=train_df, method='glm', tuneLength=20, trControl = fitControl)

# Train the model using rf

set.seed(1234); model_rf = train(Time~ .,

data=train_df, method='rf', tuneLength=20, trControl = fitControl)

# Train the model using svm

set.seed(1234); model_svmRadial = train(Time~ .,

data=train_df, method='svmRadial', tuneLength=10, trControl = fitControl)

# Train the model using naive Bayes

set.seed(1234); model_NB = train(Time~ .,

data=train_df, method='naive_bayes', tuneLength=10, trControl = fitControl)

# Train the model using Gradient Boost

set.seed(1234); model_GBM = train(Time~ .,

data=train_df, method='gbm', tuneLength=10, trControl = fitControl)

# Train the model using decision trees

set.seed(1234); model_DT = train(Time~ .,

data=train_df, method='rpart', tuneLength=10, trControl = fitControl)

## Then compare

# Compare model performances using resample()

models_compare <- resamples(list(GLM=model_glm, RF=model_rf,

NaiveBayes=model_NB,MARS=model_mars,

SVM=model_svmRadial, DT=model_DT))

# Summary of the models performances

sink("3.GEN_Training set.txt")

cat("Summary of Training using ROC metrics")

print(summary(models_compare))

sink()

# Plot ROCs

test4 <- evalm(list(GLM=model_glm, RF=model_rf,

NaiveBayes=model_NB,MARS=model_mars,

SVM=model_svmRadial, DT=model_DT),

gnames=c('glm', 'rf', 'NB', 'mars', 'svm', 'DT'),rlinethick=0.8,fsize=8,

plots='r')

tiff(file="PLOTS/3.GEN_train__ROC.tiff", unit= "in", res = 300, width = 6, height = 6)

test4$roc

dev.off()

# Draw box plots to compare models

tiff(file="PLOTS/4.GEN_train_Box_comp).tiff", unit= "in", res = 300, width = 6, height = 6)

scales <- list(x=list(relation="free"), y=list(relation="free"))

bwplot(models_compare, scales=scales)

dev.off()

#############################################################################

# TESTING #

#############################################################################

test_df = testG_df

# Predict on test. Data print to summary sheet

sink("4.GEN model testing.txt")

cat("Summary of Testing predictions using confusion matrix.\n")

cat("Reference training population = 6 baseline GEN, 6 post GEN pateints.\n")

cat("Reference testing population = 21 baseline Focal, 20 post FOCAL pateints.\n")

# model_mars

cat("MARS-model on test\n")

predicted_test <- predict(model_mars, newdata=test_df)

cat("Predicted values\n")

print(predicted_test)

print(confusionMatrix(reference = as.factor(test_df$Time), data = predicted_test,

mode='everything', positive='Y'))

cat("MARS-model on train\n")

#train

predicted_train=predict(model_mars, newdata=train_df)

print(predicted_train)

print(confusionMatrix(reference = as.factor(train_df$Time),

data = predicted_train, mode='everything', positive='Y'))

#model_glm

cat("glm model on test\n")

predicted_test <- predict(model_glm, newdata=test_df)

print(predicted_test)

print(confusionMatrix(reference = as.factor(test_df$Time), data = predicted_test,

mode='everything', positive='Y'))

#train

cat("glm model on train\n")

predicted_train=predict(model_glm, newdata=train_df)

print(predicted_train)

print(confusionMatrix(reference = as.factor(train_df$Time), data = predicted_train,

mode='everything', positive='Y'))

#model_rf

cat("rf-model on test\n")

predicted_test <- predict(model_rf, newdata=test_df)

print(predicted_test)

print(confusionMatrix(reference = as.factor(test_df$Time), data = predicted_test,

mode='everything', positive='Y'))

#train

cat("rf model on train\n")

predicted_train=predict(model_rf, newdata=train_df)

print(predicted_train)

print(confusionMatrix(reference = as.factor(train_df$Time), data = predicted_train,

mode='everything', positive='Y'))

#model_NB

cat("Naive-Bayes model on test\n")

predicted_test <- predict(model_NB, newdata=test_df)

print(predicted_test)

print(confusionMatrix(reference = as.factor(test_df$Time), data = predicted_test,

mode='everything', positive='Y'))

#train

cat("Naive-Bayes model on train\n")

predicted_train=predict(model_NB, newdata=train_df)

print(predicted_train)

print(confusionMatrix(reference = as.factor(train_df$Time), data = predicted_train,

mode='everything', positive='Y'))

#model_GBM

cat("GBM model on test\n")

predicted_test <- predict(model_GBM, newdata=test_df)

print(predicted_test)

print(confusionMatrix(reference = as.factor(test_df$Time), data = predicted_test,

mode='everything', positive='Y'))

#train

cat("GBM model on train\n")

print(predicted_train)

print(confusionMatrix(reference = as.factor(train_df$Time), data = predicted_train,

mode='everything', positive='Y'))

#model_svmRadial

cat("svmRadial model on test\n")

predicted_test <- predict(model_svmRadial, newdata=test_df)

print(predicted_test)

print(confusionMatrix(reference = as.factor(test_df$Time), data = predicted_test,

mode='everything', positive='Y'))

#train

cat("svmRadial model on train\n")

predicted_train=predict(model_svmRadial, newdata=train_df)

print(predicted_train)

print(confusionMatrix(reference = as.factor(train_df$Time), data = predicted_train,

mode='everything', positive='Y'))

#model_DT

cat("Decision Tree model on test\n")

predicted_test <- predict(model_DT, newdata=test_df)

print(predicted_test)

print(confusionMatrix(reference = as.factor(test_df$Time), data = predicted_test,

mode='everything', positive='Y'))

#train

cat("Decision Tree model on train\n")

predicted_train=predict(model_DT, newdata=train_df)

print(predicted_train)

print(confusionMatrix(reference = as.factor(train_df$Time), data = predicted_train,

mode='everything', positive='Y'))

sink()
