# Supplementary material for: Differential Transcript Expression and Alternative RNA Splicing Patterns to Differentiate Focal vs. Generalized-Onset Seizures
Source: Mol Neurobiol. 2025 Jun 11;62(10):13303–17. doi: 10.1007/s12035-025-05110-1 (PMC12433373; doi:10.1007/s12035-025-05110-1)
Supplement: Supplementary file 2 — Supplementary file2 (PDF 3078 KB) [file 12035_2025_5110_MOESM2_ESM.pdf]

Suppl. Figure 1

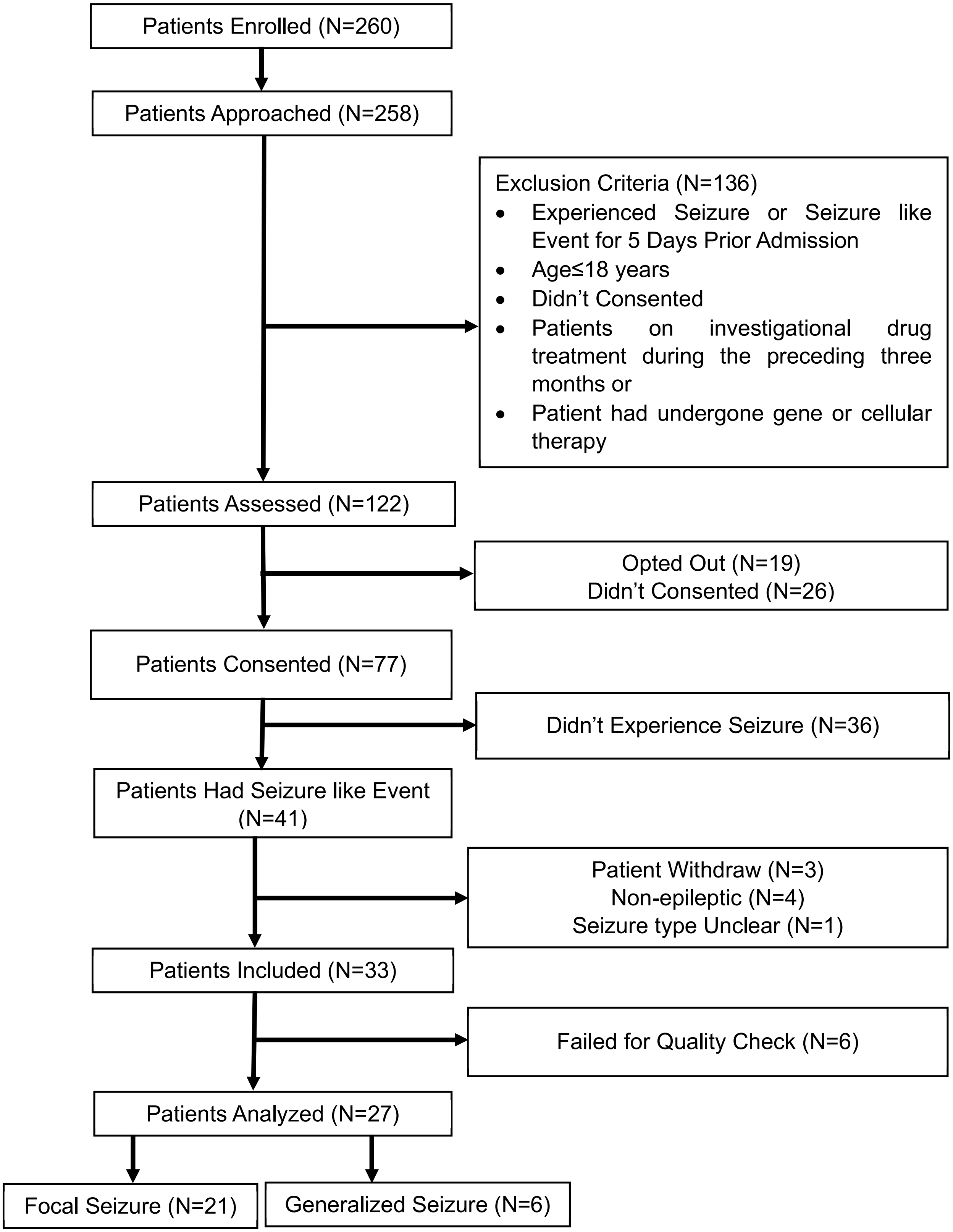

Suppl. Fig. 2

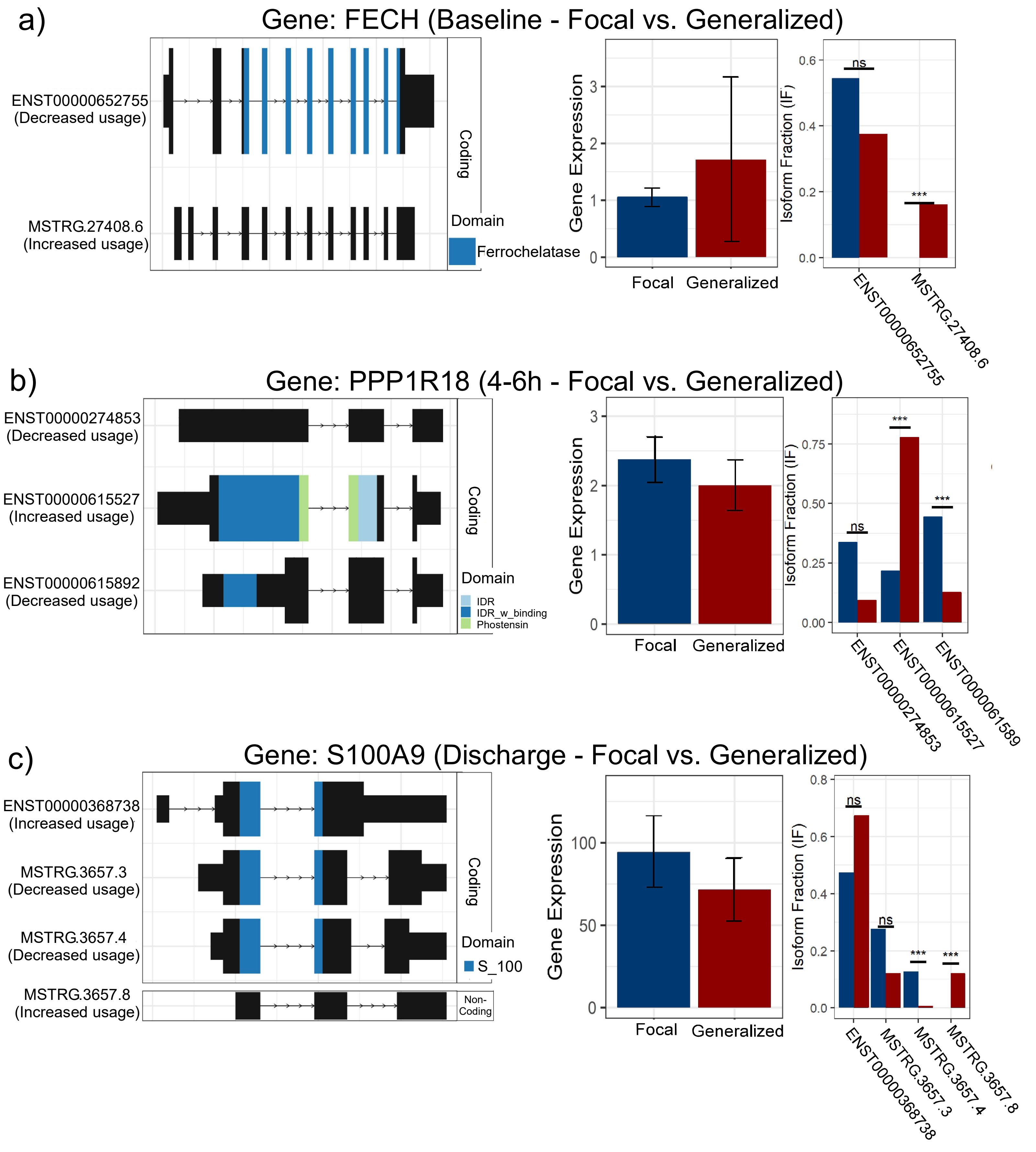
a)
Gene: FECH (Baseline - Focal vs. Generalized)
ENST00000652755
(Decreased usage)
MSTRG.27408.6
(Increased usage)
Coding
Domain
Ferrochelatase
Gene Expression
Focal
Generalized
Isoform Fraction (IF)
ns
***
ENST00000652755
MSTRG.27408.6
b)
Gene: PPP1R18 (4-6h - Focal vs. Generalized)
ENST00000274853
(Decreased usage)
ENST00000615527
(Increased usage)
ENST00000615892
(Decreased usage)
Coding
Domain
IDR
IDR_w_binding
Phostensin
Gene Expression
Focal
Generalized
Isoform Fraction (IF)
ns
***
***
ENST00000274853
ENST00000615527
ENST0000061589
c)
Gene: S100A9 (Discharge - Focal vs. Generalized)
ENST00000368738
(Increased usage)
MSTRG.3657.3
(Decreased usage)
MSTRG.3657.4
(Decreased usage)
MSTRG.3657.8
(Increased usage)
Coding
Non-Coding
Domain
S_100
Gene Expression
Focal
Generalized
Isoform Fraction (IF)
ns
ns
***
***
ENST00000368738
MSTRG.3657.3
MSTRG.3657.4
MSTRG.3657.8

Suppl. Fig. 3

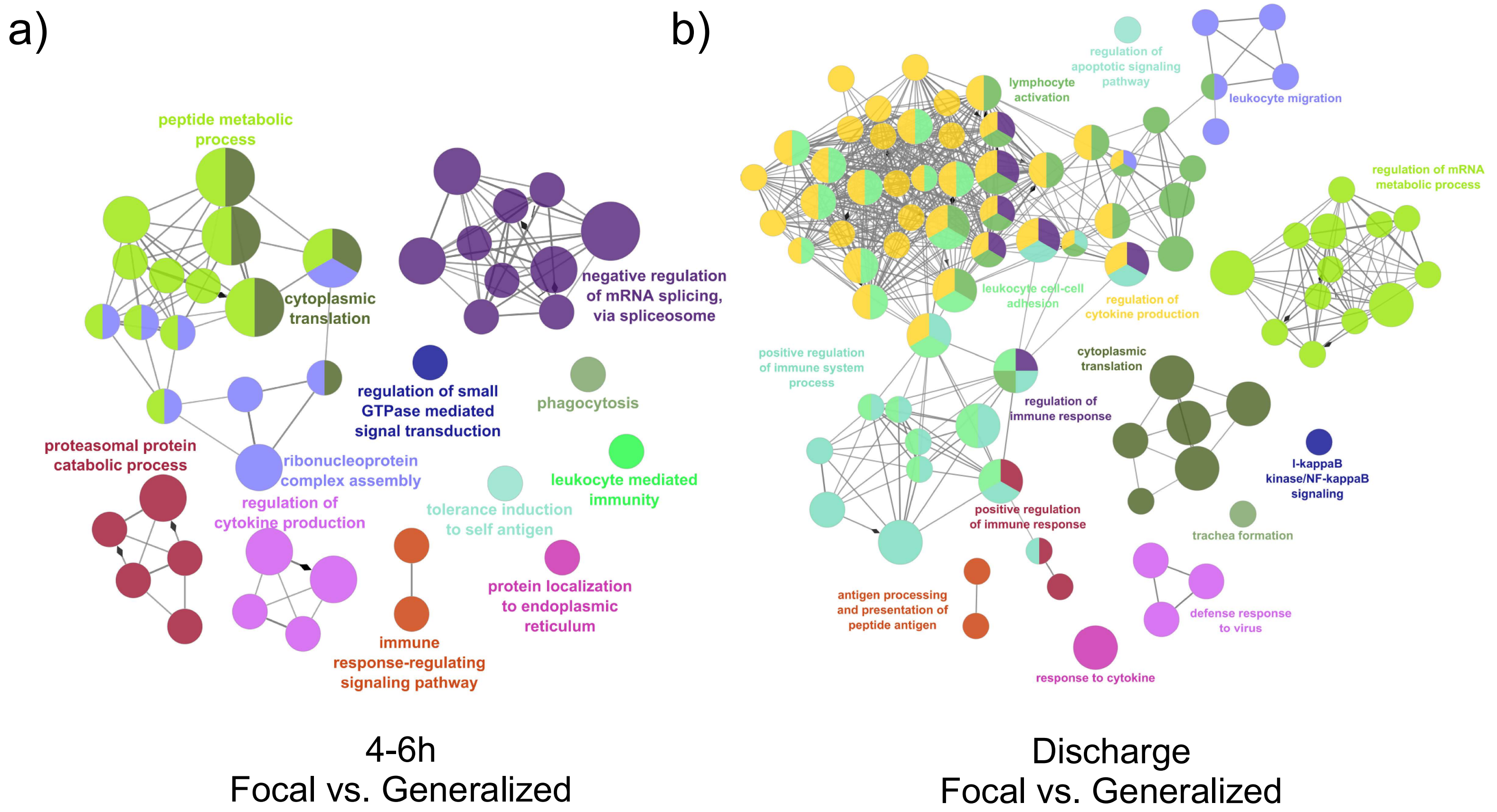
a)
peptide metabolic process
cytoplasmic translation
negative regulation of mRNA splicing, via spliceosome
regulation of small GTPase mediated signal transduction
phagocytosis
proteasomal protein catabolic process
ribonucleoprotein complex assembly
regulation of cytokine production
leukocyte mediated immunity
tolerance induction to self antigen
protein localization to endoplasmic reticulum
immune response-regulating signaling pathway
4-6h
Focal vs. Generalized
b)
regulation of apoptotic signaling pathway
lymphocyte activation
leukocyte migration
regulation of mRNA metabolic process
leukocyte cell-cell adhesion
regulation of cytokine production
positive regulation of immune system process
cytoplasmic translation
regulation of immune response
I-kappaB kinase/NF-kappaB signaling
positive regulation of immune response
trachea formation
antigen processing and presentation of peptide antigen
defense response to virus
response to cytokine
Discharge
Focal vs. Generalized

Suppl. Fig. 4

a) Focal Seizure Training (Discharge)

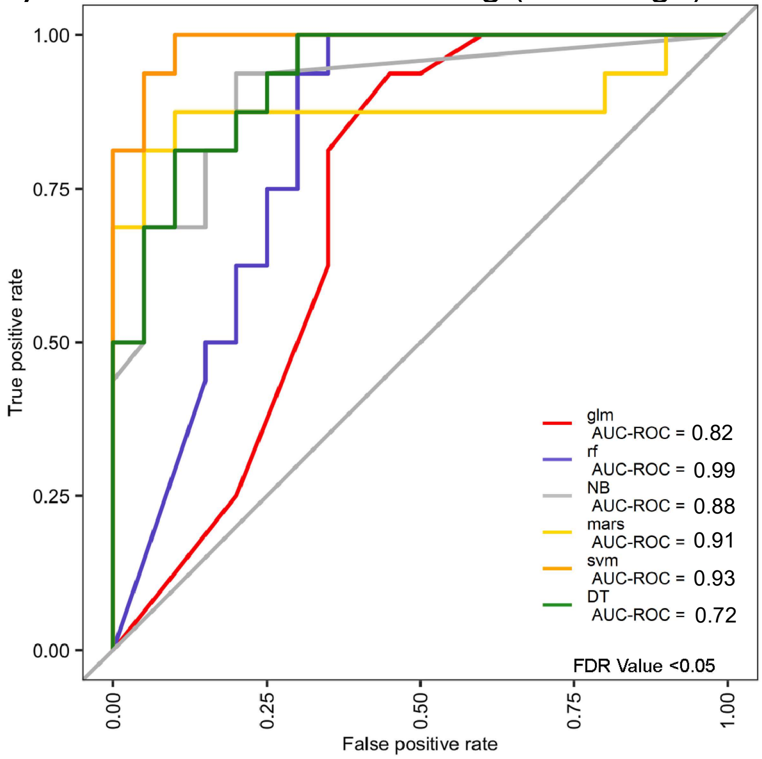

Train Focal Seizure Performance Metrics

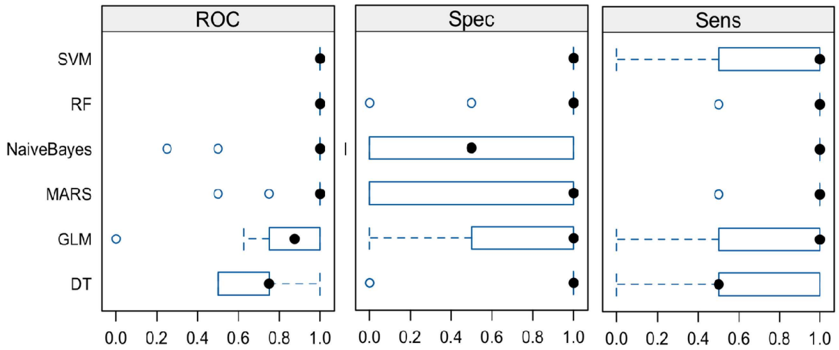

Confusion Matrices: Tested on Generalized Seizure Data Set

Predicted (Focal Seizure)	Actual Data (Generalized Seizure): YES	Actual Data (Generalized Seizure): NO
YES	2	1
NO	4	5

b) Generalized Seizure Training (Discharge)

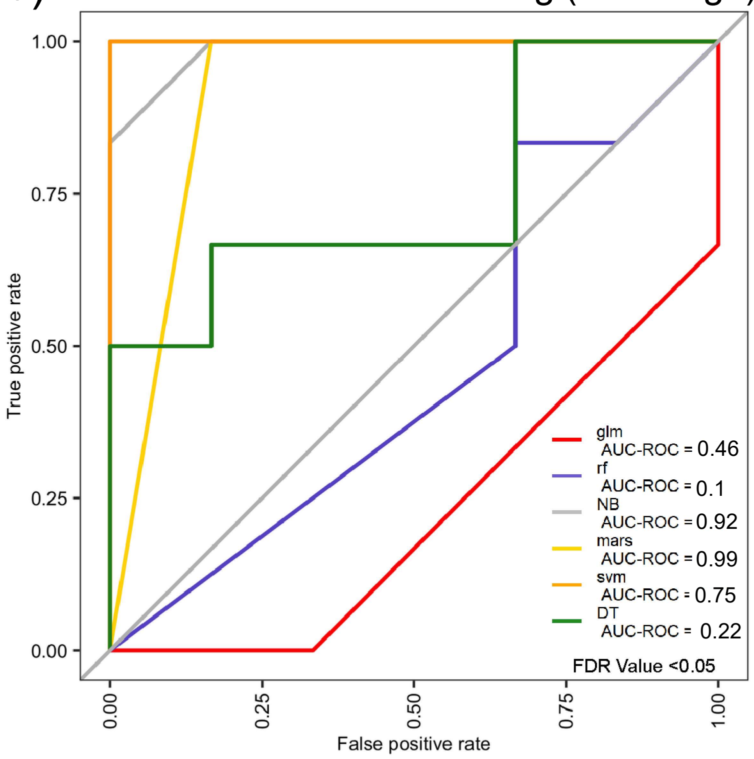

Train Generalized Seizure Performance Metrics

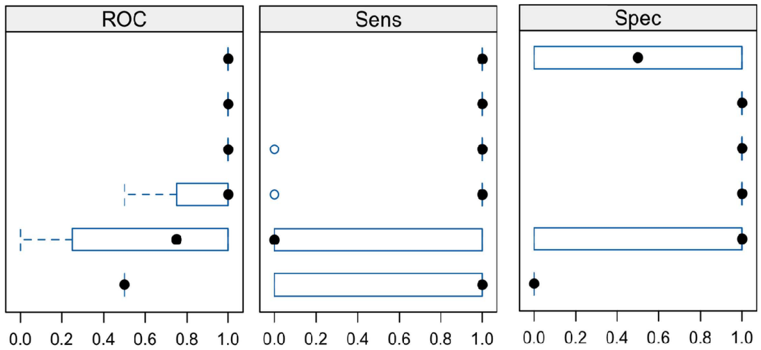

Confusion Matrices: Tested on Focal Seizure Data Set

Predicted (Generalized Seizure)	Actual Data (Focal Seizure): YES	Actual Data (Focal Seizure): NO
YES	7	13
NO	9	7
